# Human ex vivo 3D bone model recapitulates osteocyte response to metastatic prostate cancer

**DOI:** 10.1038/s41598-018-36424-x

**Published:** 2018-12-19

**Authors:** Saba Choudhary, Poornema Ramasundaram, Eugenia Dziopa, Ciaran Mannion, Yair Kissin, Lucas Tricoli, Christopher Albanese, Woo Lee, Jenny Zilberberg

**Affiliations:** 1 Department of Biomedical Engineering, Chemistry and Biological Sciences, Stevens Institute of Technology, Hoboken, NJ USA; 2 Center for Discovery and Innovation, Hackensack University Medical Center, Nutley, NJ USA; 3 Department of Pathology, Hackensack University Medical Center, Hackensack, NJ USA; 4 Insall Scott Kelly Institute for Orthopedics and Sports Medicine, New York, NY USA; 5 Hackensack University Medical Center, Hackensack, NJ USA; 6 Lenox Hill Hospital, New York, NY USA; 7 Lombardi Comprehensive Cancer Center, Georgetown University, Washington, DC USA; 8 Department of Chemical Engineering and Materials Science, Stevens Institute of Technology, Hoboken, NJ USA

## Abstract

Prostate cancer (PCa) is the second leading cause of cancer deaths among American men. Unfortunately, there is no cure once the tumor is established within the bone niche. Although osteocytes are master regulators of bone homeostasis and remodeling, their role in supporting PCa metastases remains poorly defined. This is largely due to a lack of suitable *ex vivo* models capable of recapitulating the physiological behavior of primary osteocytes. To address this need, we integrated an engineered bone tissue model formed by 3D-networked primary human osteocytes, with conditionally reprogrammed (CR) primary human PCa cells. CR PCa cells induced a significant increase in the expression of fibroblast growth factor 23 (FGF23) by osteocytes. The expression of the Wnt inhibitors sclerostin and dickkopf-1 (Dkk-1), exhibited contrasting trends, where sclerostin decreased while Dkk-1 increased. Furthermore, alkaline phosphatase (ALP) was induced with a concomitant increase in mineralization, consistent with the predominantly osteoblastic PCa-bone metastasis niche seen in patients. Lastly, we confirmed that traditional 2D culture failed to reproduce these key responses, making the use of our *ex vivo* engineered human 3D bone tissue an ideal platform for modeling PCa-bone interactions.

## Introduction

Prostate cancer (PCa) is the second leading cause of cancer deaths among American men^1,2^. While initially an androgen-driven disease, PCa morbidity and mortality is primarily the result of metastases that have become androgen-independent^3,4^. Bone is the preferred site for PCa metastases, and currently no curative treatments exist once the tumor is established within this niche^5–7^. Due to the poor prognosis and increased morbidity associated with PCa metastases, a better understanding of the complex interactions of the tumor with the bone microenvironment is imperative. Osteocytes are master regulators of bone remodeling^8–10^. Recent studies have shown that osteocytes may influence PCa progression in bone metastasis, but their role remains poorly defined^11,12^. Investigating the crosstalk between osteocytes and cancer cells is critical in identifying potential therapeutic targets to halt tumor progression and prevent metastasis to bone.

Unfortunately, progress in gaining a more complete understanding of the interactions between disseminated tumor cells and bone has been impeded by the lack of relevant models. Many of the current tumor microenvironment platforms are not only costly, but also do not accurately recapitulate the human disease, leading to inaccurate predictions of the efficacy and safety of drug outcomes in humans^13–17^. Furthermore, commonly used cell lines do not entirely recapitulate the heterogeneity of primary PCa cells^18–21^.

The rapid establishment and maintenance of long-term *ex vivo* primary cultures from patient-derived PCa tumor samples has historically been extremely difficult. The recent development of the organoid^22,23^ and the conditional reprogramming technologies has greatly enhanced the repertoire of primary human prostate available^24^. The CR technology is a rapid two dimensional culture platform based on co-culturing primary cells with irradiated-3T3 mouse fibroblasts (or in conditioned media from these cells) in the presence of a Rho-associated protein kinase inhibitor. The CR platform has been widely applied to both normal and malignant biopsied samples from many epithelial tissues^24–29^. Importantly, conditionally reprogrammed (CR) cells have the potential to differentiate when placed *in vivo* or under permissive *in vitro* culture conditions, making them an important resource for translational research^27^.

Tissue engineered three-dimensional (3D) models are an ideal platform to investigate the crosstalk between bone and cancer cells^30^. We and others have shown that 3D culture systems support the growth and maturation of osteocytes *in vitro*, which is not possible using traditional two-dimensional (2D) methods^31–36^. Specifically, we showed that primary human osteocytes can be assembled with 20–25 µm microbeads and cultured in a microfluidic perfusion device to replicate the lacunocanalicular structure and functions of human bone tissue^35,37^. Most recently, we established that hypoxic 3D culture of human primary osteocytic cells enhanced osteocyte phenotype *ex vivo* while enabling the spontaneous formation of an osteoblastic monolayer that resembles the endosteal layer^38^. This single cell layer primarily comprised of osteoblasts and localized at the interface between the bone marrow and bone, is critical in bone metastasis since it represent the site where disseminated tumor cells interact with the bone and become dormant and drug resistance until tumor reactivation and progression^39^.

In this study, we carried out a targeted investigation of the changes induced by PCa cells on osteocytes. For the first time, we integrated 3D bone tissue modeling with CR cells to characterize the bone microenvironment of metastatic PCa, using exclusively primary human cells.

## Results

### PCa cells compromise the morphology of engineered 3D bone tissues

**Figure 1:**
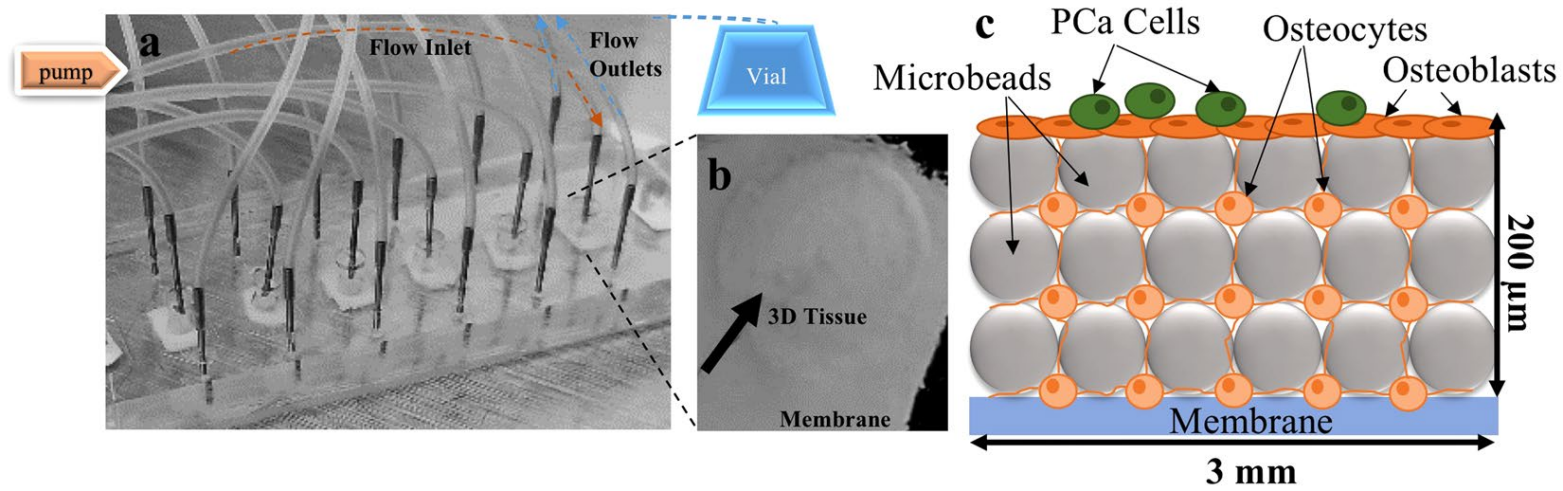
Microfluidic perfusion device for engineering 3D bone tissues. (**a**) Actual device, containing bone tissue constructs in the central chamber with medium flowing into one inlet fed by a syringe pump and exiting through two outlets carrying effluent to a collection vial. Tissues were constructed using BCP microbeads and primary human osteocytes assembled at a 1:1 ratio (**b**) Harvested 3D tissue. (**c**) Schematic illustration of the tissue constructs showing the location of the PCa cells, osteoblasts forming the endosteum, and the spatial distribution of osteocytes and microbeads (not drawn to scale).

We formed 3D bone tissues in microfluidic perfusion devices (Fig. 1) by culturing primary human osteocytes with BCP microbeads for 14 days. Comparisons of dendrite length and cell-cell distance (Table 1)^40^ between hypoxic and normoxic (from our previous studies^38^) 3D bone tissues revealed that hypoxia induces profound changes in the 3D structure of osteocytic cells by producing cells with prolonged dendrites.

**Table 1:** Dendrite Length Measurements in Histological Sections of 3D Bone Tissues.

3D Tissue	Dendrite length (µm)	Cell to Cell distance (µm)
Hypoxia*	19.24 ± 5.88	24.37 ± 4.42
Normoxia	14.20 ± 2.74	19.57 ± 3.78

*p < 0.001 compared to normoxia. Average of at least 15 randomly selected sites in 3D tissue sections.

**Figure 2:**
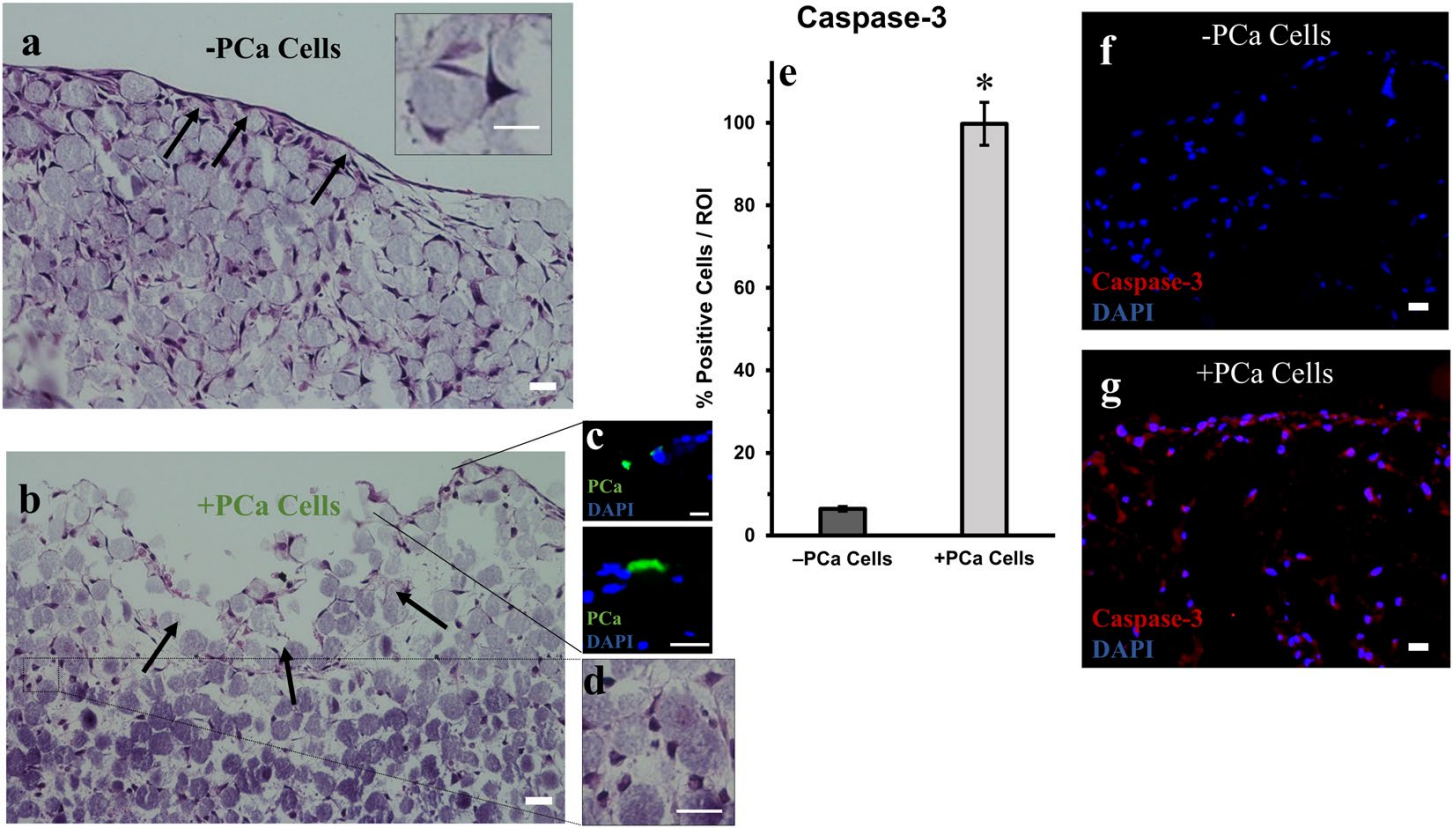
Histology sections of the engineered 3D bone tissues. Representative H&E staining of vertical 3D tissue sections (**a)** −PCa cells (control cultures without PCa cells), showing an intact endosteal layer (black arrows) and (**b**) +PCa cells (co-cultured with PCa cells) showing compromised tissue (black arrows). (**c**) Sections were stained with pan-cytokeratin to identify PCa cells (green). (**d**) Representative image showing the atypically rounded morphology of osteocytes throughout the tissue when cultured with PCa cells. (**e**) Quantification of active caspase-3 immunofluorescence staining (*p < 0.01 compared to −PCa controls). Representative images of immunofluorescence staining for active caspase-3 in tissues (**h**) −PCa cells (**g**) +PCa cells. ROI = region of interest. Scale bars = 20 µm.

PCa cells were introduced to the system and cultured for another 4 days before harvesting the tissue (Fig. 1b). The morphology of the tissues was observed by H&E staining (Fig. 2). In the tissue without PCa cells (−PCa cells, Fig. 2a), the osteocytes were well spread out, with dendrites protruding to neighboring cells (inset) and the endosteal layer (characterized previously^38^) was intact (Fig. 1a, black arrows).

Conversely, the 3D tissues co-cultured with PCa cells were compromised (+PCa cells, Fig. 2b–d). The introduction of PCa cells significantly impacted the endosteal layer (Fig. 2b, black arrows) and the underlying tissue structure as indicated by the rounded morphology of osteocytes (Fig. 2d). In addition, we observed that PCa cells were adherent to the endosteal surface (Fig. 2c) and while PCa cells were not detected in the interior region of the tissues, co-culture with PCa cells significantly affected the integrity of the underlying reconstructed bone as evidenced by the significant increase (p < 0.01) in active caspase-3 staining in the bone tissues exposed to PCa cells (+PCa cells, Fig. 2e,g). Very little active caspase-3 staining was found in the control tissues (−PCa cells, Fig. 2f). The tissue with PCa stained highly positive for active caspase-3 throughout, from the endosteal surface to the inner region of the 3D bone tissue (+PCa cells, Fig. 2g).

### Wnt signaling inhibitors are altered in osteocytes of 3D bone tissues exposed to PCa cells

**Figure 3:**
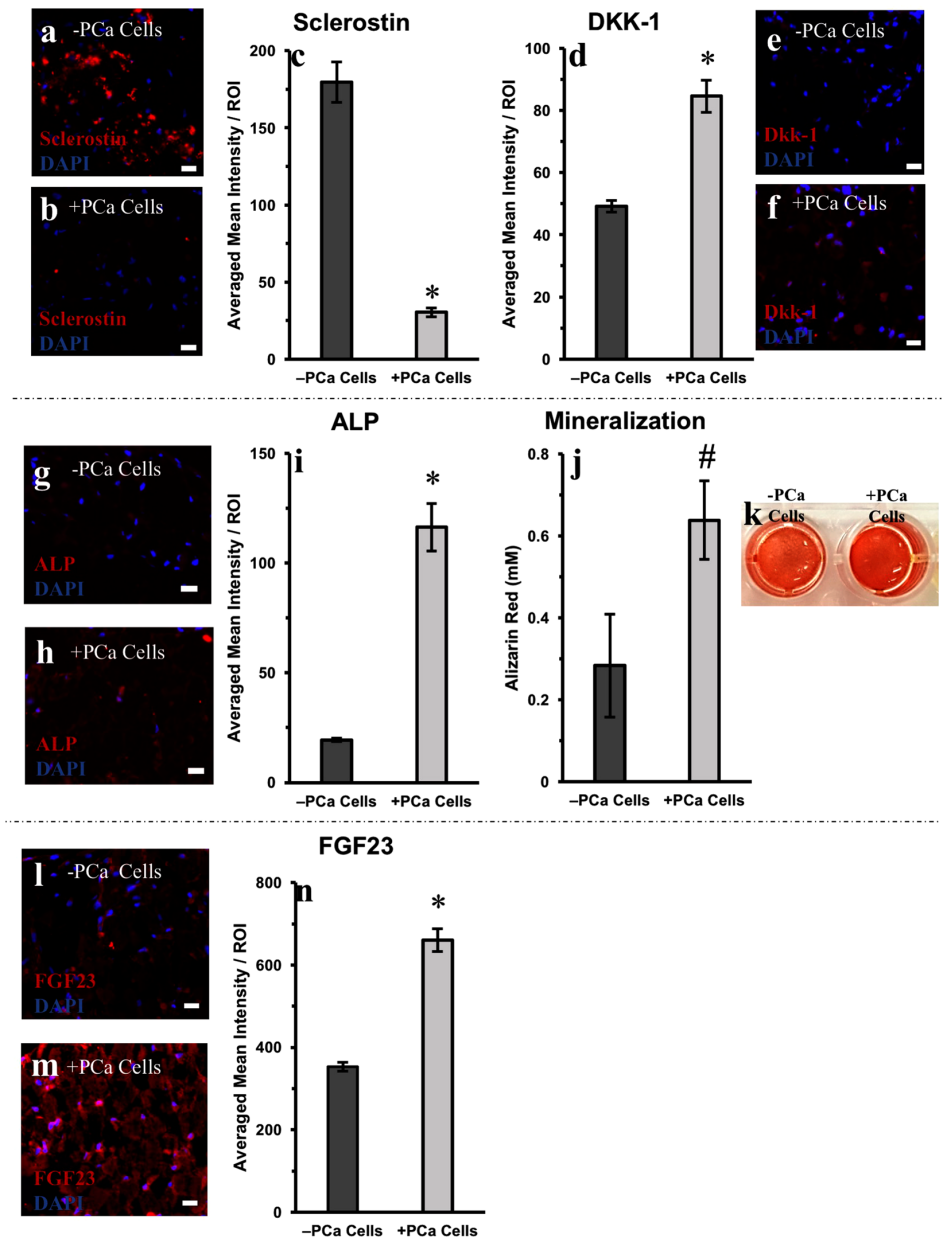
Staining and quantification of key osteo-related markers in engineered 3D bone tissues cultured with and without PCa cells. Representative images of sclerostin staining in 3D tissue sections (**a**) −PCa cells (control cultures without PCa cells) and (**b**) +PCa cells (co-cultured with PCa cells). Quantification of (**c**) sclerostin immunofluorescence staining and (**d**) Dkk-1 immunofluorescence staining. Representative images of Dkk-1 staining in 3D tissue sections (**e)** −PCa cells (**f**) +PCa cells. Representative image of ALP staining in 3D tissue sections (**g**) −PCa cells and (**h**) +PCa cells. Quantification of (**i**) ALP immunofluorescence staining and (**j**) mineralization. (**k**) Representative image of extracted Alizarin Red S from 3D tissues –PCa cells (left) or +PCa cells (right). Representative images of FGF23 staining in 3D tissue sections (**l**) −PCa cells and (**m**) +PCa cells. (**n**) Quantification of FGF23 immunofluorescence staining. *p < 0.01 and ^#^p = 0.02 compared to −PCa controls. ROI = region of interest. Scale bars = 20 µm.

In order to evaluate the role of osteocytes in bone remodeling induced by PCa cells, tissue sections were stained for Wnt signaling inhibitors – sclerostin and dickkopf-1 (Dkk-1). Sclerostin was widely expressed by the osteocytes in the 3D tissues in the absence of PCa cells (−PCa cells, Fig. 3a). Interestingly a six-fold decrease (p < 0.01) in sclerostin was observed in osteocytes that were co-cultured with PCa cells (+PCa cells, Fig. 3b,c). While Dkk-1 was expressed by osteocytes in the 3D tissue (−PCa cells, Fig. 3e), exposure to PCa cells resulted in a significant 1.7-fold increase in this protein (p < 0.01, Fig. 3d,f).

### Osteoblastic characteristics are found in 3D bone tissues cultured with PCa cells

We next investigated the osteoblastic nature of PCa bone metastasis by examining the expression of alkaline phosphatase (ALP), an indication of bone-forming osteoblastic activity. We found that ALP significantly increased (p < 0.01) with the introduction of PCa cells (+PCa cells, Fig. 3h,i), with a concomitant increase in mineralization (Fig. 3j,k). Alizarin Red S staining (which stains for calcium deposition) was 1.6-fold higher in the 3D tissues co-cultured with PCa cells (p = 0.02).

### Osteocytes increase fibroblast growth factor 23 expression in 3D bone tissues with PCa

An important emerging target in bone metastasis is fibroblast growth factor 23 (FGF23), which is expressed by mature osteocytes^41^. We used our 3D bone tissue model to assess the levels of FGF23 expression by osteocytes, in the presence and absence of PCa cells. As seen in Fig. 3l, FGF23 was found to be expressed throughout the tissue sections, however the introduction of PCa cells resulted in a significant, nearly two-fold increase (p < 0.01), in FGF23 expression by osteocytes (Fig. 3m,n).

### Gene expression analyses corroborate immunofluorescence trends

**Figure 4:**
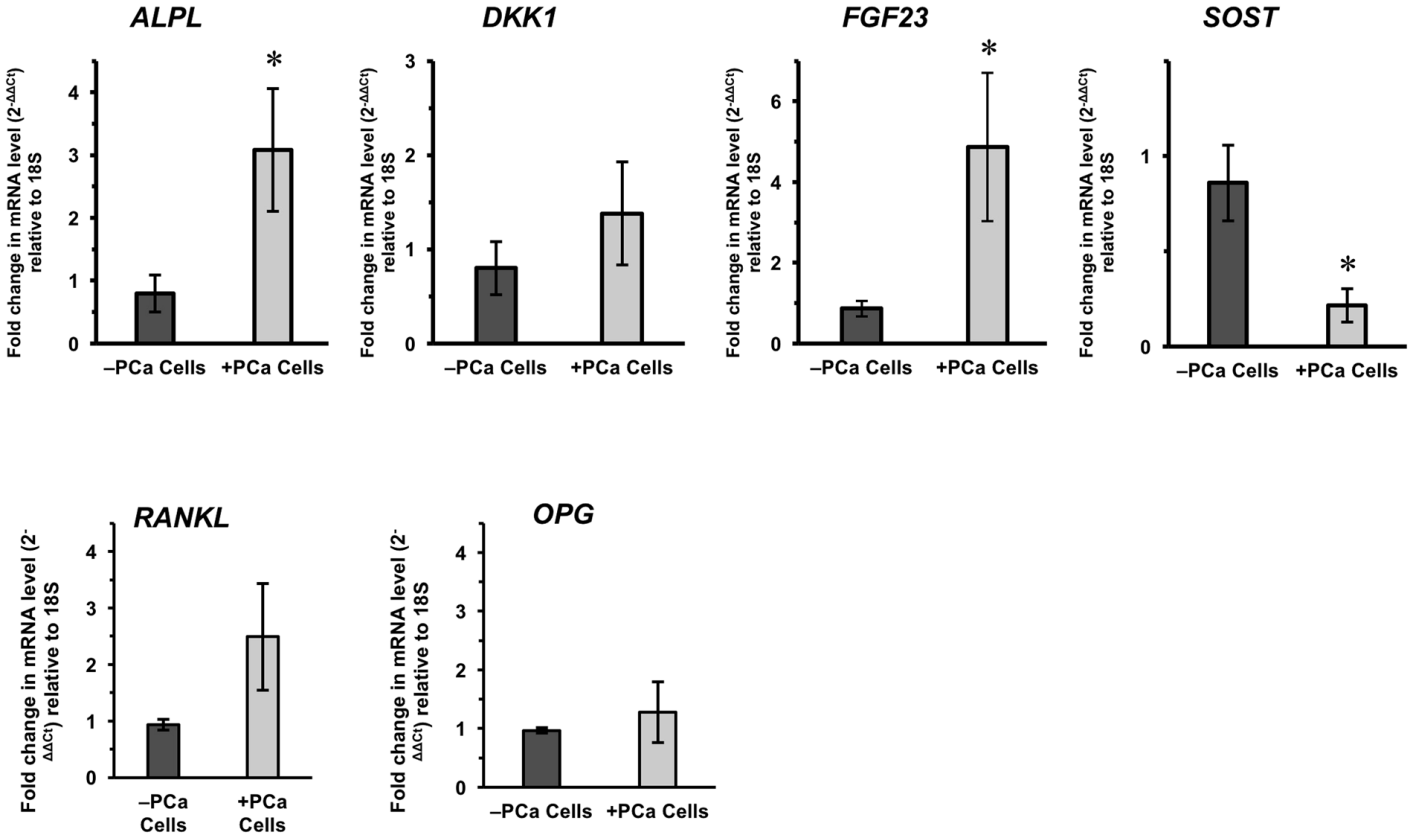
Comparison of osteo-related gene expression of engineered 3D bone tissues. Fold change was calculated following the ΔΔCT method to compare gene expressions between 3D tissues cultured without PCa cells (−PCa cells) versus 3D tissues cultured with PCa cells (+PCa cell) as 2^(−ΔΔCt)^. *p = 0.04 compared to −PCa controls. ND = none detected.

To verify our immunofluorescence staining and quantification of protein expression, we analyzed gene expressions in the engineered 3D bone tissues using qRT-PCR. The trends we observed in immunofluorescence analyses were confirmed with qRT-PCR (Fig. 4). Overall, ALPL (the gene for ALP) and FGF23 gene expression were significantly increased, whereas *SOST* (encoding sclerostin) significantly decreased (p = 0.04). Although not statistically significant, DKK1 expression was also increased. In addition, we examined the gene expressions of RANKL and OPG. 3D bone tissues with PCa cells showed an increasing trend in RANKL mRNA levels while OPG remained unchanged.

### 2D Co-cultures do not recapitulate key expressional changes in osteocytes

**Figure 5:**
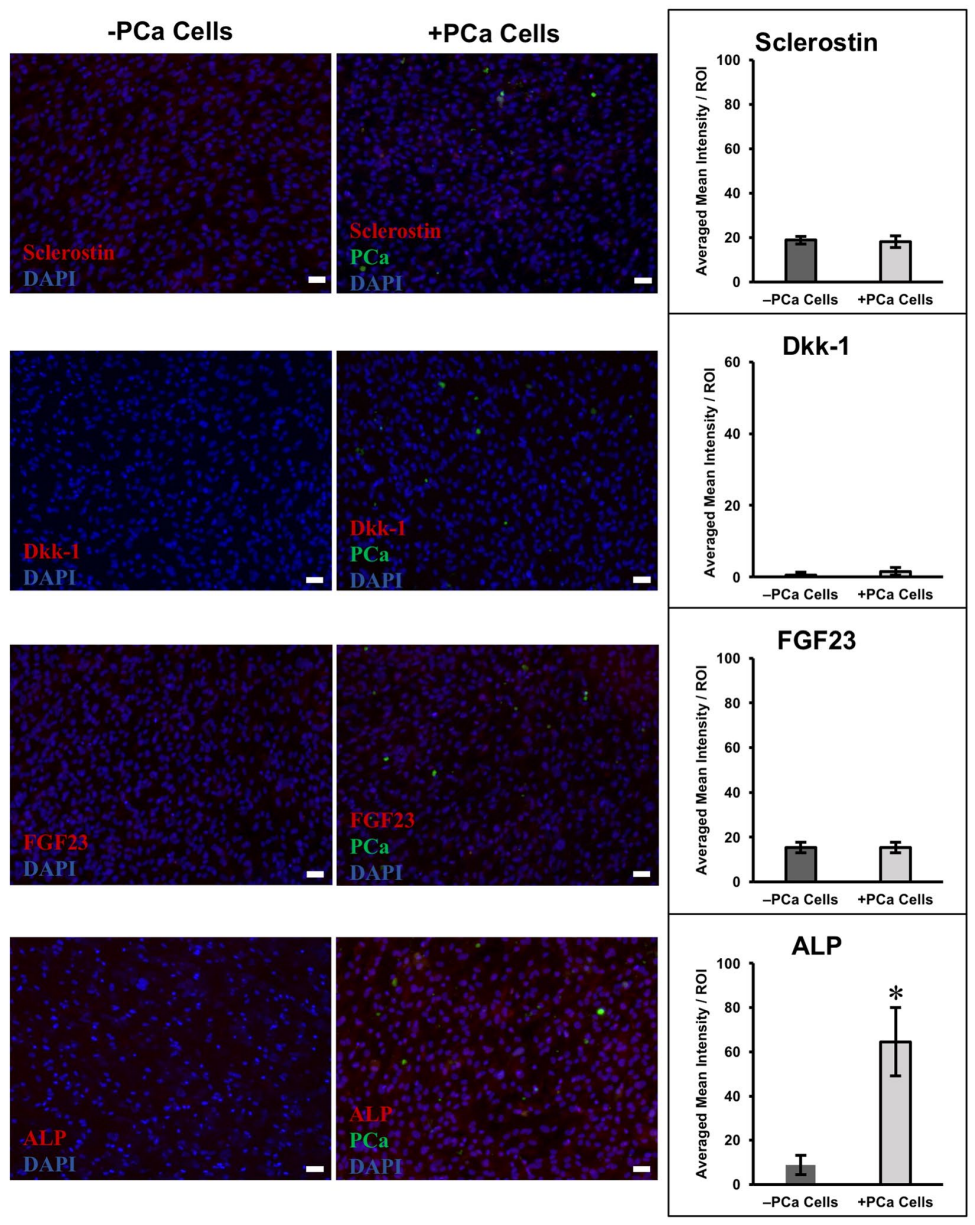
Immunofluorescence staining and quantification of 2D cultures of primary human osteocytic cells with and without PCa cells. Representative images of immunofluorescence staining of sclerostin, Dkk-1, FGF23, and ALP in 2D cultures without PCa cells (−PCa cells, left images) or with PCa cells (+PCa cells, right images). Bar graphs show quantification of immunofluorescence staining. *p < 0.01 compared to −PCa controls. Scale bars = 50 µm.

To highlight the importance of 3D culture, we evaluated the interaction of PCa and osteocytic cells in 2D. We cultured osteocytic cells (5 × 10^4^) in traditional 2D culture plates for 14 days and then introduced PCa cells (2 × 10^4^) for 4 days, as we did for 3D experiments. Expression of FGF23, sclerostin, and Dkk-1 were relatively low and no significant difference was observed between cultures (Fig. 5). Differences were observed in ALP expression, where ALP significantly increased (p < 0.01) with the introduction of PCa cells into the 2D cultures. Mineralization in 2D cultures was also analyzed (Supplementary Fig. 1), and the change was not statistically significant.

### CR PCa cells do not exhibit extensive osteomimetic behavior

PCa cells have been shown to have the ability to mimic bone by displaying osteo-associated phenotypic and genotypic signatures^42^. To characterize the osteomimetic behavior of the CR PCa cells used in our study, we carried out gene expression analyses to verify that the genetic changes observed in the 3D bone tissues were in fact coming from the bone cells. The PCa cells were cultured for 4 days in their CR cells conditioned medium (see methods section), to evaluate any inherent osteogenic gene expression the cells may possess, and compared to genotypic expression upon culturing in a 1:1 medium mixture containing CR cells conditioned medium and osteogenic differentiation medium collected from 3D bone tissue cultures (i.e., the effluent collected in vials shown in Fig. 1a). PCa cells did not express any of the osteogenic markers evaluated in this study, aside from SOST at very low levels (Supplementary Fig. 2). Furthermore, this expression was unchanged when cells were cultured in the 1:1 medium.

### CR PCa cells are viable under osteogenic conditions

In order to confirm that CR PCa cells could be cultured with osteocytes, we had first assessed the viability of CR PCa cells in a 1:1 mixture of CR cells conditioned medium and osteogenic differentiation medium over a period of 4 days. As seen in Supplementary Fig. 3, the 1:1 medium had no adverse effect on cell viability.

We next measured CR PCa cells adherence to a confluent layer of primary human osteocytic cells. CFSE-labeled PCa cells were allowed to interact with osteocytes for 4 days. PCa cells exhibited mild adhesion to the osteocytic cells – approximately half of the PCa cells remained adherent while the remainder were easily removed by rinsing with PBS (Supplementary Fig. 4).

### CR PCa cells proliferate when cultured in 3D bone tissues

PCa cells were pre-labeled with the cell proliferation dye eFluor 670 prior to introduction to the 3D bone tissues, to track the presence of these cells in the cultures. After a 4-day period, cultures were collected and analyzed by flow cytometry. Because we observed that PCa cells were moderately adherent (Supplementary Fig. 4), we examined both the supernatant of the 3D cultures and dissociated the tissues for further examination. The results from these experiments showed that PCa cells proliferated and remained within the 3D bone tissues, (~6% of total number of gated cells were still eFluor 670+ after the culture period); (Supplementary Fig. 5) however, cells in the supernatant were found to be not viable (data not shown).

## Discussion

Traditional 2D culture models fail to recapitulate key microenvironmental factors which are critical for proper drug development and evaluation^43^, and more biologically relevant tissue-engineered 3D models are needed to bridge this gap^30^. In this study, we developed an all-human model to investigate metastatic PCa interactions with osteocytes by integrating our previously developed an characterized *ex vivo* reconstructed 3D bone tissue with CR PCa cells obtained from the lymph node of a PCa patient. Although no bone metastatic CR lines were available to conduct the current study, we chose CR PCa3 cells due to the fact that they are: (1) metastatic and (2) easily expandable. Importantly, culturing any CR cell line in our model can allow us to capture the behavior of these cells when interacting with osteocytes, as a means of determining the potential for bone metastasis and to develop better treatments for those patients whose cells display high affinity towards bone.

Using this model we showed, for the first time to the best of our knowledge, that the expression of osteocytic FGF23, an emerging drug target in cancer and other ailments^44,45^, increased significantly in the presence of PCa cells. The broader implementation of this approach has the potential to complement, and perhaps replace various costly and difficult-to-implement animal models^13–17^, while also reducing the limitations associated with the use of conventional metastatic prostate cancer cell lines (e.g. PC3, DU145, LNCaP) which suffer from significant genetic perturbations following decades in culture and at best, are each representative of only one patients’ tumor^46^.

Previous 3D bone metastasis models of PCa failed to specifically pinpoint the effects of cancer cells on osteocytes^47,48^. A systematic review of *in vitro* 3D bone metastasis models was recently carried out by Salamanna *et al*.^30^. In this review, the authors appropriately note that 3D *in vitro* models can be designed to capture different physiological elements and stages of metastatic progression. However, they also pointed out that there is no ideal *in vitro* model that can mimic all of these *in vivo* events. Therefore, *in vitro* models represent biomimetic snapshots that can be developed to answer specific questions such as: “What kind of 3D *in vitro* model should be used to model particular aspects of human disease?”, “Are these models able to catalyze the development of new therapeutic interventions?”, or “How much the proposed model can help in elucidating the mechanisms at the basis of bone invasion and metastasis?” In this review, three different standardized analyses were carefully performed to compared different models: (1) 3D device-assisted assembly models of bone metastasis, (2) 3D matrix-assisted assembly models of bone metastasis, (3) 3D direct bone tumor cell contact models of bone metastasis. In contrast to the approximately 23 systems examined, our model uniquely combined all of the following unique features: (1) primary human osteocytes (not used in any other metastatic model), (2) hypoxic conditions, and (3) integration with CR cells as opposed to use of conventional cell lines. Furthermore, while valuable information on overall trends of key biomarkers in bone metastasis were provided, the other studies did not address whether the expressional changes detected were derived specifically from osteocytes as we have done, for the first time, here.

Sieh *et al*.^49,50^, also recognized the need for using primary human osteoblast instead of modified human osteoblastic cell lines to improved the biomimetic nature of the construct with respect to native human bone tissues. The authors found that the integrity of the human osteoblasts was crucial to allow a dynamic intercellular communication in the co-culture model. However, in this model, no osteocytes or hypoxia were used to recapitulated the bone microenvironment and the PC3 and LNCaP lines were utilized as surrogates of human PCa.

Lastly, Salamanna *et al*.^48^, recently reported on the use of a humanized 3D *in vitro* model where human bone fragments were *ex vivo* preserved in a rotating bioreactor maintained under hypoxic conditions and breast cancer cell lines were added to investigate their effects as a function of bone origin (i.e., pre and postmenopausal patients). In this innovative work the authors reported key cytokine production differences between healthy and osteoporotic bone in response to tumor cells, substantiating the need for using patient-derived samples to understand the complex interactions of tumor cells with bone. Although the specific contribution of cell type (i.e., osteoblasts, osteocyte, breast cancer cells, etc.) was not reported, it is likely that it could also be used to explore cell-specific cytokine production through immunohistochemistry, as we did in our study.

Evaluating the contribution of osteocyte-specific markers – FGF23, sclerostin, Dkk-1 – is important, as these proteins have gained attention as potential targets in cancer treatments. FGF23 has been shown to promote PCa progression and has been postulated to increase the formation of bone metastasis^51,52^. PCa cells have also been shown to secrete and express receptors for FGF23^52,53^, which may likely contribute to increased circulating FGF23 levels in patients. However, it remains to be investigated whether bone-secreted FGF23 itself is a contributor of circulating FGF23, and how it could potentially play a role in PCa chemotaxis to this niche^52,53^.

Immunofluorescence staining of tissue sections showed that osteocyte FGF3 expression was significantly increased in the presence of PCa cells (Fig. 3n). To further validate our findings, we performed gene expression analysis and confirmed that there was a significant increase in *FGF23* in 3D bone tissues cultured with PCa cells (Fig. 4). We established that the increase was solely from osteocytes by verifying that the CR PCa cells used did not express *FGF23* (Supplementary Fig. 2).

We also noted that PCa cells did not invade the 3D tissues and were found sporadically at the endosteal layer (Fig. 2c). To validate the presence of these cells in the 3D tissues, we designed a parallel experiment were PCa cells were pre-labeled with eFluor 670, a cell proliferation dye, to track the cells in the cultures (Supplementary Fig. 5). Although the seeding density was ~1:5 PCa to osteocytes, flow cytometric analyses conducted showed that the dissociated tissues were constituted by only ~6% PCa cells after a 4-day culture period, likely due to the following factors: (1) osteocytes expanded more than PCa cells (note that these cells were cultured for 14 days before introduction of tumor cells), (2) PCa cells lost eFluor 670 due to proliferation, (3) not all the PCa cells were maintained in the culture because these particular CR cells appeared to be mildly adherent (Supplementary Fig. 4) to bone cells, and (4) tissue integrity was affected by PCa cells, reducing the necessary matrix for these cells to adhere. That notwithstanding, these results and factors corroborate that PCa cells remained in the tissues and could also account for their scarce numbers in the immunofluorescence images.

Osteocytes secrete Wnt inhibitors, sclerostin and Dkk-1^54^. These proteins can promote osteolytic lesions in PCa by inhibiting the Wnt signaling pathway associated with bone formation and homeostasis. Previous studies have suggested opposing roles of sclerostin and Dkk-1 in bone metastasis^54^. We found that osteocytes had decreased expression of sclerostin and increased levels of Dkk-1, when in the presence of CR PCa cells. The greater decrease in sclerostin may favor osteoblastic lesions; however, Dkk-1 may act as a “molecular switch” towards osteolytic lesions as previously suggested^54^.

Immunofluorescence staining (Fig. 3) showed decreased sclerostin expression in the osteocytes within the 3D tissues cultured with PCa cells concurrent with a significant decrease in *SOST* expression compared to control tissues (−PCa cell, Fig. 4). Of note, CR PCa cells expressed minimal basal levels of *SOST* expression, which remained unchanged when the cells were cultured in the 1:1 medium prepared with the effluent of 3D bone tissue cultures (Supplemental Fig. 2).

Importantly while we did not directly assess β-catenin expression or localization; a measurement of bone functioning^55,56^, both RANKL and OPG expression can be controlled by Wnt/β-catenin signaling. OPG has been shown to be a direct target of β-catenin transcriptional activation, and loss of β-catenin leads to decreased OPG expression and increased osteoclast activity. β-Catenin-deficient osteoblasts have also been documented to express higher RANKL expression. Conversely, activation of Wnt/β-catenin signaling by stabilizing β-catenin increased OPG expression and bone formation^57^. Our data indicates an increasing tendency in the RANKL mRNA expression suggesting a potential modulation of β-catenin.

Tissue ALP expression increased significantly in the presence of PCa cells, with a concomitant increase in mineralization. However, despite the new bone formation, the 3D bone tissues exposed to PCa cells were unhealthy, resulting in tissue fragility and more challenging post-culture processing. This is consistent with literature showing that PCa induces predominantly osteoblastic or mixed lesions, and induces the formation of low quality bone that is prone to fracture^58^. In contrast, other studies have found the PC3 cell line, which is widely used as a model of metastatic PCa, gives rise to primarily osteolytic lesions^59^.

Following the introduction of PCa cells, FGF23, Dkk-1 and sclerostin protein expressions remained unchanged in 2D hypoxic cultures (Fig. 5), suggesting that 3D culture conditions are needed to maintain an osteocytic phenotype, as we previously observed^38^. We were only able to identify a significant increase in ALP when PCa cells were introduced into 2D culture (Fig. 5). However, this increase was not associated with increased bone mineralization (Supplementary Fig. 1), which was an order of magnitude less than that observed in 3D (Fig. 3j). These results further suggest that, unlike 2D cultures, our 3D platform captures key microenvironmental features necessary to carry out pathophysiologically relevant studies of cancer-bone interaction.

PCa is a heterogeneous disease with variability not only amongst cancer cells within a patient, but large variability across patients^60^. Our 3D platform could therefore be used for patient-specific applications. Our analyses were done using a relatively low seeding density of PCa to bone cells to mimic dissemination of tumor cells to the bone niche. Despite using low number of PCa cells, significant changes in the osteocytes within the 3D bone model were observed, suggesting that this model system is amenable to relative small sample sizes while still providing important information regarding the tumor-bone interactions. In addition, the CR PCa cells exhibited a robust affinity for bone, further suggesting that the 3D system may enable patient-specific applications, such as with small amounts of circulating tumor cells to study bone metastasis.

In conclusion, this study serves as a successful proof-of-concept that our 3D bone model has the potential of providing new insight into the crosstalk between bone and disseminated/metastatic tumor cells. We anticipate that this platform, which utilizes primary human osteocytes and primary prostate cancer cells, indefinitely propagated using the conditional reprogramming system, can be used as a transformative means to: (1) evaluate therapeutic targets in a personalized manner, (2) develop and screen new treatments that particularly target the bone niche, and (3) study the effects of tumor-bone interactions as a major mediator of microenvironmental-induced drug-resistance^61^.

## Materials and Methods

### Primary human osteocytes

Primary human osteocytic cells were isolated as previously described^38^. Discarded bone samples were collected with informed consent from orthopedic surgery patients, de-identified, and processed in accordance with an approved protocol by the Institution Review Board (IRB) of Hackensack Meridian Health. Bone samples were cut into small bone chips and subjected to a series of digestions alternating the use of collagenase and EDTA. After 7 digestions, the bone chips were plated onto collagen-coated 6-well tissue culture plates and osteocytic cells were allowed to migrate out of the bone chips for 8 days. The osteocytic cells from 3 patients, previously characterized^35,38^, were kept frozen in liquid nitrogen for use in this study.

### Cell culture

Primary human osteocytic cells were cultured in α-MEM supplemented with 10% FBS and 1% P/S. All cultures were maintained at 37 °C in a sterile, humidified, 5% CO_2_ incubator. For experiments using osteogenic differentiation medium, 3 mM β-glycerophosphate and 50 µg/mL L-ascorbic acid was added to the base medium. All experiments were conducted under hypoxic conditions, in a Heracell Vios 160i incubator (Thermo Fisher) at 4% oxygen and all culture medium was pre-incubated in 4% oxygen for 24 h prior to use. In experiments where 3D bone tissues were cultured with CR PCa (+PCa cells), perfused medium consisted of a 1:1 mixture of each cell type’s medium.

### Culture of CR prostate cancer cells

The PCa cells utilized in this study were isolated from the lymph node of a patient (PCa3) and propagated as organoids previously characterized^22^. Tricoli and Albanese (authors of this paper) at the Lombardi Comprehensive Cancer Center received the organoid cultures from Gao *et al*.^22^, through an established collaboration. The organoids were enzymatically dispersed, and cultured as CR cells as previously described and characterized^24,27,29^. Briefly, the PCa cells were propagated in 2D using conditioned medium prepared with feeder fibroblast cells and a 3:1 (v/v) mixture of F-12 nutrient mixture and Dulbecco’s modified Eagle’s medium, 5% FBS, and 5 µmol/L of Rho-associated kinase (ROCK) inhibitor Y-27632 (Santa Cruz Biotechnology). These cells readily stained positive for pan-cytokeratin (Supplementary Fig. 6), an epithelial marker commonly used to identify PCa tumor cells (see below for staining methods).

From karyotyping and basic molecular analysis, the cells established from the PCa3 organoid using the conditionally reprogramming method demonstrate consistency with the original PCa3 prostate organoid line established. Additionally, as mentioned, the CR methodology for maintaining primary cell cultures using this method are well documented^28,29^. The cells utilized were maintained in the standard CR conditions in 2D, so there is no cell death due to disaggregation.

### Engineering of 3D bone tissue in perfusion culture

The microfluidic culture devices were made with polydimethylsiloxane (PDMS) with 8 culture chambers, as described previously^34^. Soft lithography was used to create 200 µm thick hexagonal patterns of 6 mm × 12 mm with a central 3 mm diameter culture chamber. Each culture chamber was secured with a 200 µm thick Microweb filter membrane (Millipore). The PDMS was then bonded to a glass slide. The device was sterilized by washing all chambers and microfluidic channels with 70% isopropyl alcohol.

The 3D human bone tissue was reconstructed in the center of the culture chamber, using collagen-coated biphasic calcium phosphate (BCP) microbeads of 20–25 µm diameter (CaP Biomaterials). Primary human osteocytic cells were combined with BCP microbeads at a 1:1 ratio. To each culture chamber, 3D tissues were constructed by seeding a mixture of 1 × 10^5^ cells and 1 × 10^5^ beads. Perfusion was initiated by connecting the inlet port with polyethylene tubing that served differentiation medium to the central culture chamber with a syringe pump (KD Scientific), at a rate of 1 µL/min. Effluent medium was collected via polyethylene tubing connecting the outlets to a collection vial (Fig. 1).

Culture devices were prepared and placed under hypoxic (4% oxygen) conditions. Tissues were allowed to grow for 14 days and then 2 × 10^4^ CR PCa cells were introduced into the chamber for another 4 days, to mimic the presence of disseminated cancer cells in the bone niche. Only medium was added to control 3D bone tissue chambers (−PCa cells). The medium to all the tissues was changed to a 1:1 mixture of the CR cells conditioned medium and the osteogenic differentiation medium. For some experiments where PCa cells were monitored using flow cytometry, the cells were pre-labeled with 2.5 μM of the cell proliferation dye eFluor 670 (eBioscience) as per manufacture’s instructions.

### Histology

Engineered 3D tissues were harvested, rinsed in PBS, and fixed in 4% PFA. Fixed samples were sent to the Histology Core Facility at the New Jersey Medical School of Rutgers University for paraffin embedding and sectioning. Vertical tissue sections 10 µm thick were stained with hematoxylin and eosin (H&E, Sigma-Aldrich) and examined for distribution and morphology. Dendrite length was determined by measuring the distance from the cell body to the end of the dendrite projection.

### Immunofluorescence staining

After deparaffinization and rehydration, samples were subjected to heat-induced antigen retrieval in EDTA buffer, pH 8.5 (Sigma-Aldrich). Samples were permeabilized with 0.1% Triton X-100 for 10 min and blocked with 3% BSA (w/v) for 1 h at room temperature. Samples were stained with rabbit anti-human sclerostin (1:10, ab75914), rabbit anti-human ALP (1:10, ab75699), rabbit anti-human active caspase-3 (1:100, ab2302), rabbit anti-human FGF23 (1:50, ab192497), or rabbit anti-human Dkk-1 (1:50, ab61034) overnight at 4 °C, followed by incubation with a secondary stain (1:100 TRITC-conjugated goat anti-rabbit IgG, ab50598) for 1 h at room temperature and counterstained with DAPI containing mounting medium (Fluoroshield with DAPI, Sigma). To identify PCa cells, samples were stained with mouse anti-human pan cytokeratin (1:100, ab86734) followed by secondary staining with Alexa-Fluor 488-conjugated goat anti-mouse IgG (1:100, ab150113). All antibodies were purchased from Abcam.

Fluorescence was quantified using image analysis software (NIS-Elements, Nikon). For each sample, 10 regions of interest (ROI) were randomly selected, each containing 8–10 cells. Mean intensity was quantified for each ROI and averaged for all samples of the same group.

### Cell viability

PCa cells were seeded onto 96-well plates. Cells were cultured either in PCa cell medium only or in a 1:1 mixture of the PCa medium with osteogenic differentiation medium. Cell viability was assessed using a Live/Dead viability/cytotoxicity kit according to the manufacturer’s instructions (Thermo Fisher Scientific). Briefly, the staining solution was prepared in PBS with 6 µM Calcien-AM (live stain) and 6 µM Ethidium homodimer-1 (dead stain) and incubated at 37 °C for 30 min. The cells were visualized under a fluorescence microscope; live cell bodies fluoresced green after being excited by blue light, whereas dead cells’ nuclei fluoresced red after excitation with green light. Cells were counted in 10 random fields of view for each sample and averaged.

### Cell adhesion

Primary human osteocytic cells were seeded onto 24-well plates and allowed to reach confluence. PCa cells were labeled with carboxyfluorescein succinimidyl ester (CFSE, 2 µM, Invitrogen) and seeded into each well to allowed them to interact with the osteocytic cells for 4 days. The cells were then rinsed with PBS 3 times. The CFSE-labeled PCa cells were imaged before and after rinsing. Cells were counted in 25 random fields of view and averaged to calculate cell density before and after the rinse.

### 2D co-culture experiments

Human primary osteocytic cells were seeded onto 24-well plates for each assessment. After growing for 14 days, PCa cells were labeled with CFSE (2 µM, Invitrogen) and then seeded into the wells. No PCa cells were introduced to control cultures. After 4 days, the cells were fixed and prepared for immunofluorescence staining as described above.

### RNA isolation, cDNA preparation, and qRT-PCR

Total RNA was isolated using Purelink RNA Mini kit (Ambion), following the manufacturer’s instructions. Complementary DNA (cDNA) was prepared by reverse transcription using 1 µg of RNA as described previously^38^. 20 µL qPCR reaction mixtures were prepared using 2 µL cDNA, 1 µL of 20x Taqman primer, 10 µL of 2x Taqman master mix, and 7 µL water. The quantitative PCR fast assay was carried out on a StepOnePlus (Applied Biosystems, CA, USA). The following amplification cycle was repeated 40 times: 95 °C, 20 sec; 90 °C, 1 sec; 60 °C, 20 sec. The comparative ΔΔCt method was used to determine the fold-change in gene expression between the −PCa controls and + PCa groups using 18 S as the endogenous control. Gene expressions for *ALPL* (encoding alkaline phosphatase, Hs01029144_m1), *DKK1* (encoding dickkopf-1, Hs00183740_m1), *FGF23* (encoding fibroblast growth factor 23, Hs00221003_m1), and *SOST* (encoding sclerostin, Hs00228830_m1) were analyzed using indicated Taqman primers (Thermo Fisher Scientific).

### PCa Osteomimetic behavior assessment

PCa cells were seeded onto 24-well plates and allowed to grow for 4 days. One set of cells were grown in regular PCa growth medium. A second set of cells were grown in a 1:1 mixture of CR cells conditioned medium with osteogenic conditioned medium – the medium collected as the effluent from the engineered 3D bone tissues. Cells were harvested after 4 days and assessed for osteogenic gene expressions – *SOST*, *DKK1*, *ALPL*, *FGF23* – as described above using qRT-PCR.

### Mineralization

After samples were harvested and fixed with 4% PFA, Alizarin Red S staining and quantification assay (ScienCell) were used according to the manufacturer’s instructions. Briefly, samples were incubated for 15 min with staining solution and then washed 3x. The stain was extracted and absorbance was measured at 405 nm. A standard curve was used to quantitate the concentration of Alizarin Red S that was extracted from samples.

### Statistical analysis

Comparisons between −PCa control and +PCa experimental groups were made using two-tailed Student’s t-test. Follow-on gene expressions were analyzed using a one-tailed Student’s t-test. A p < 0.05 was considered statistically significant. For 3D histological immunofluorescence studies n = 5 tissue constructs, for qRT-PCR n = 2, and for all other experiments n = 3 per group. Data are reported as average ± standard deviation.

## Electronic supplementary material

Supplementary Information

## Data Availability

The datasets generated in this study are available from the corresponding author on reasonable request.
