# Supplementary material for: Human ex vivo 3D bone model recapitulates osteocyte response to metastatic prostate cancer
Source: Sci Rep. 2018 Dec 19;8:17975. doi: 10.1038/s41598-018-36424-x (PMC6299475; doi:10.1038/s41598-018-36424-x)
Supplement: Supplementary file 1 — Supplementary Information [file 41598_2018_36424_MOESM1_ESM.pdf]

Human ex vivo 3D bone model recapitulates osteocyte response to metastatic prostate cancer

Saba Choudhary,[1] Poornema Ramasundaram,[2] Eugenia Dziopa,[2] Ciaran Mannion,[3] Yair Kissin,[4,5,6] Lucas Tricoli,[7] Christopher Albanese,[7] Woo Lee,[8*] Jenny Zilberberg,[2#*]

[1]Department of Biomedical Engineering, Chemistry and Biological Sciences, Stevens Institute of Technology, Hoboken, NJ USA

[2]Department of Biomedical Research, Hackensack University Medical Center, Hackensack, NJ, USA

[3]Department of Pathology, Hackensack University Medical Center, Hackensack, NJ, USA

[4]Insall Scott Kelly Institute for Orthopedics and Sports Medicine, New York, NY, USA

[5]Hackensack University Medical Center, Hackensack, NJ, USA

[6]Lenox Hill Hospital, New York, NY, USA

[7]Lombardi Comprehensive Cancer Center, Georgetown University, Washington D.C., USA

[8]Department of Chemical Engineering and Materials Science, Stevens Institute of Technology, Hoboken, NJ USA

*Equal author contribution
#Corresponding author

Jenny Zilberberg, Ph.D.

Associate Scientist

Department of Biomedical Research

Hackensack University Medical Center

40 Prospect Avenue

Hackensack, NJ 07601

Phone: 551-996-4547

Email: Jenny.Zilberberg@hackensackmeridian.org

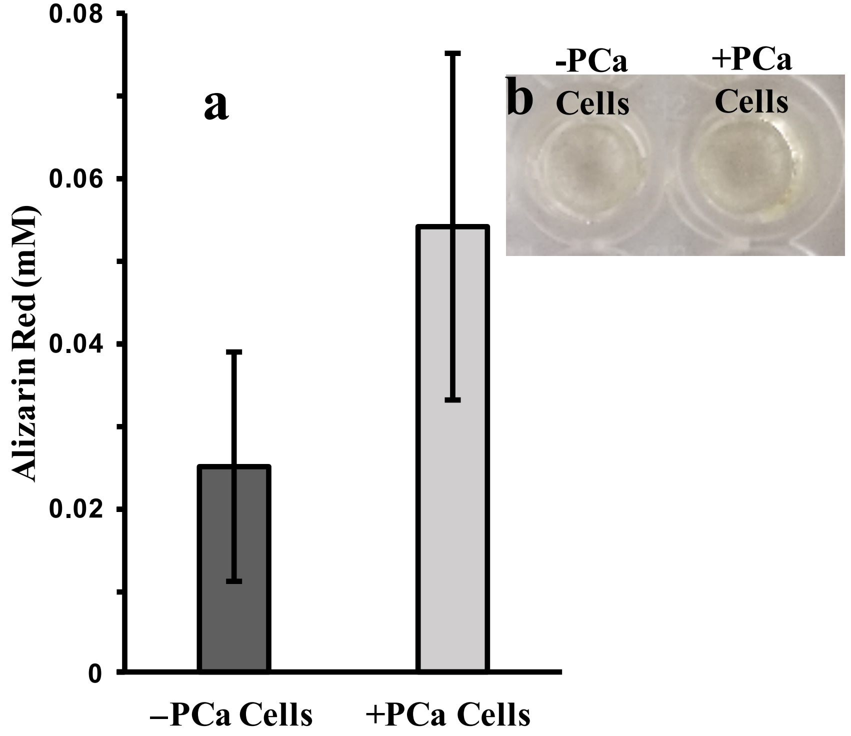

Supplementary Figure 1: Mineralization of primary human osteocytic cells in 2D culture. (a) Quantification of mineralization in 2D cultures of osteocytes with no PCa cells and with PCa cells. (b) Representative image of extracted Alizarin Red S from 2D cultures of osteocytes with no PCa cells (left) and with PCa cells (right).

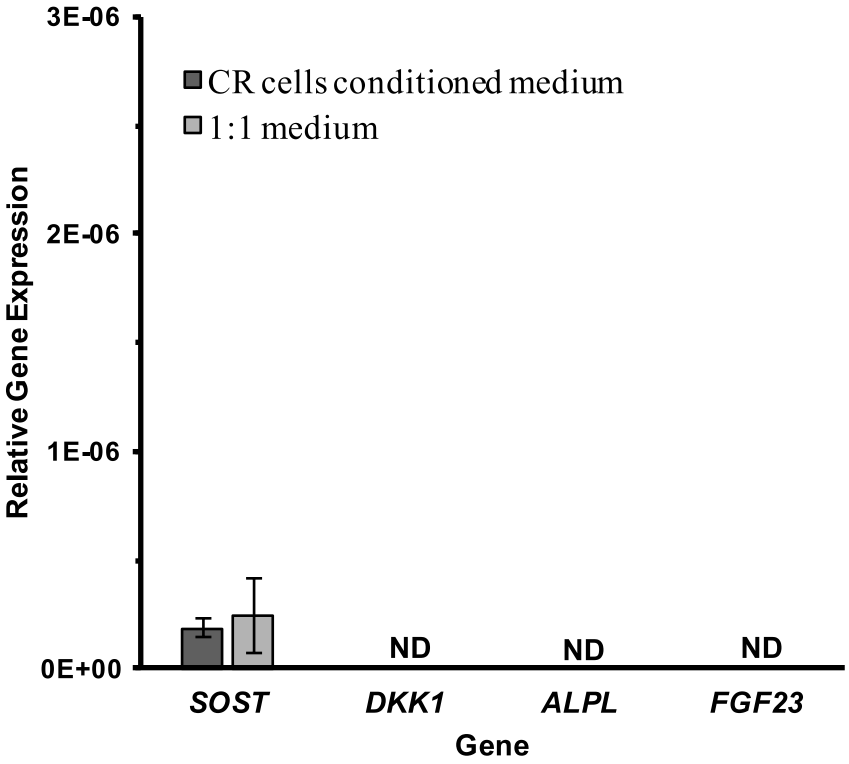

Supplementary Figure 2: Relative gene expression of PCa cells cultured in CR cells conditioned medium or in a mixture of 1:1 medium containing conditioned osteogenic medium collected as the effluent from 3D osteocyte cultures.

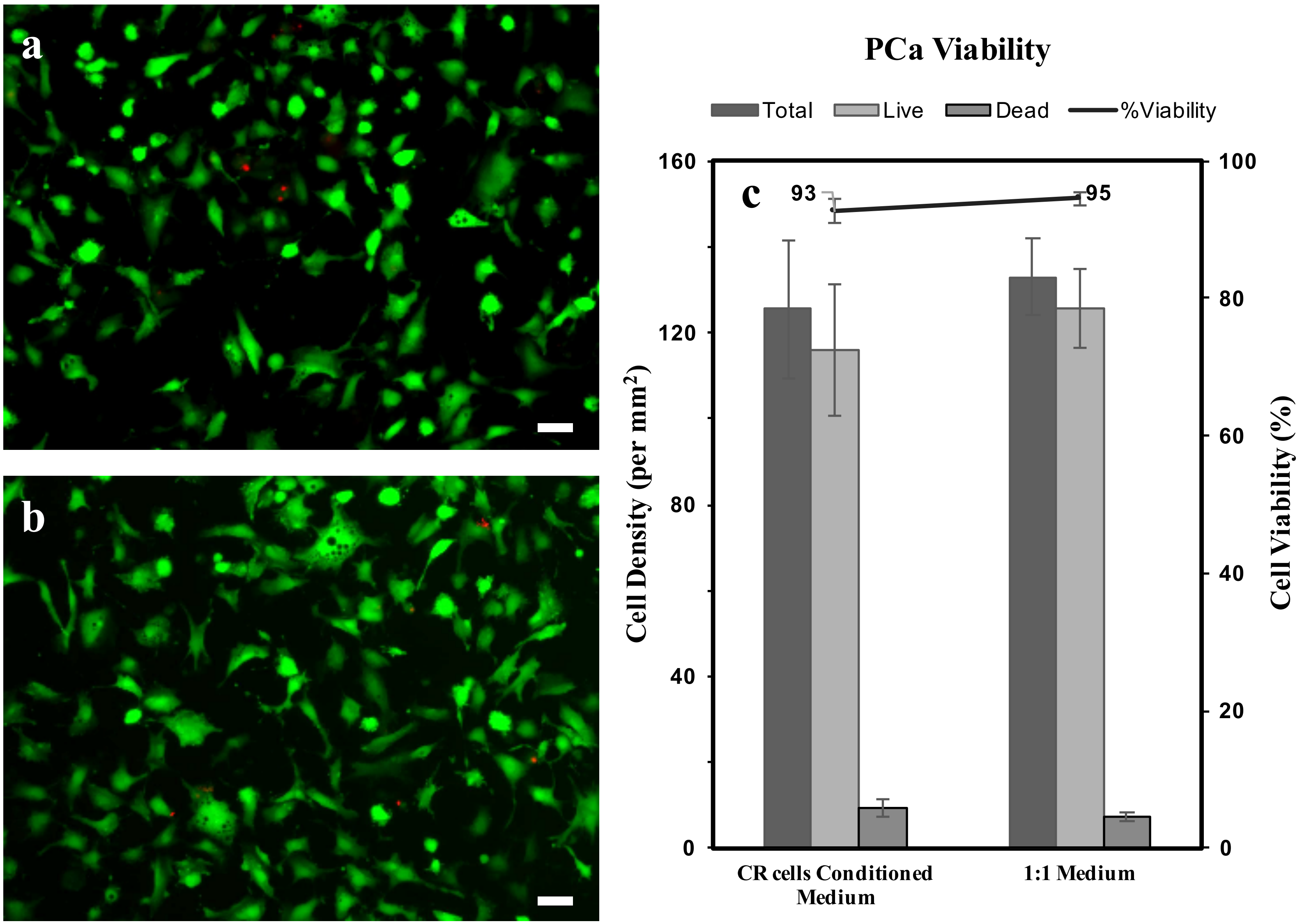

Supplementary Figure 3: PCa viability under osteogenic co-culture environment. Representative images of live (green) and dead (red) PCa cells cultured in (a) CR cells conditioned medium and (b) 1:1 mixture of CR cells conditioned medium and osteogenic differentiation medium. (c) Quantification of total, live, dead cells and % viability of PCa cells under different culture conditions. Scale bars = 50 µm.

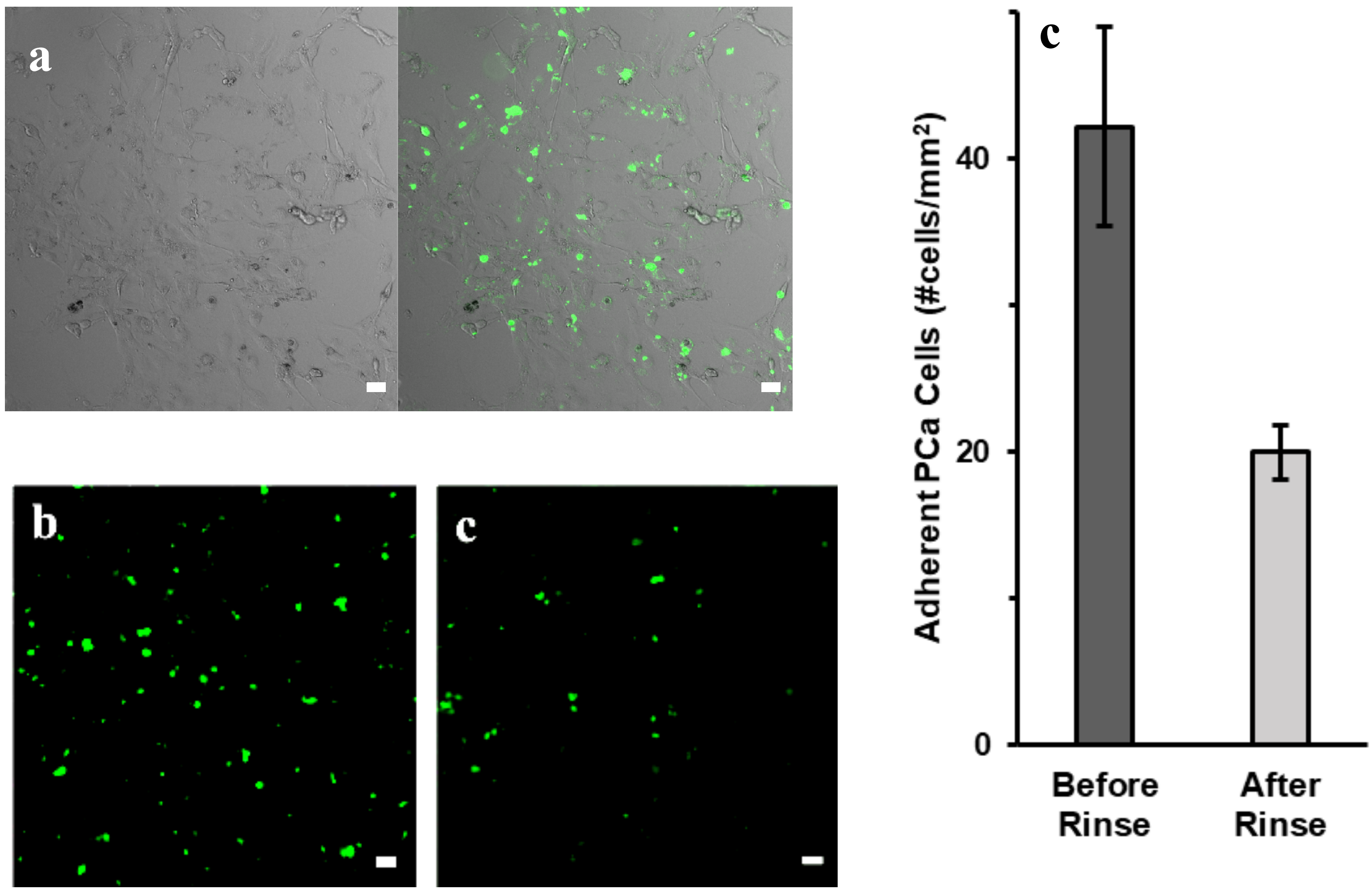

Supplementary Figure 4: Adhesion of CFSE-labeled PCa cells to human primary osteocytic cells. Representative images of PCa cells (green) at (a) 24 h, and after 4 days (b) before rinsing, and (c) after rinsing. (c) Quantification of PCa cell density before and after rinsing. Scale bars = 50 µm.

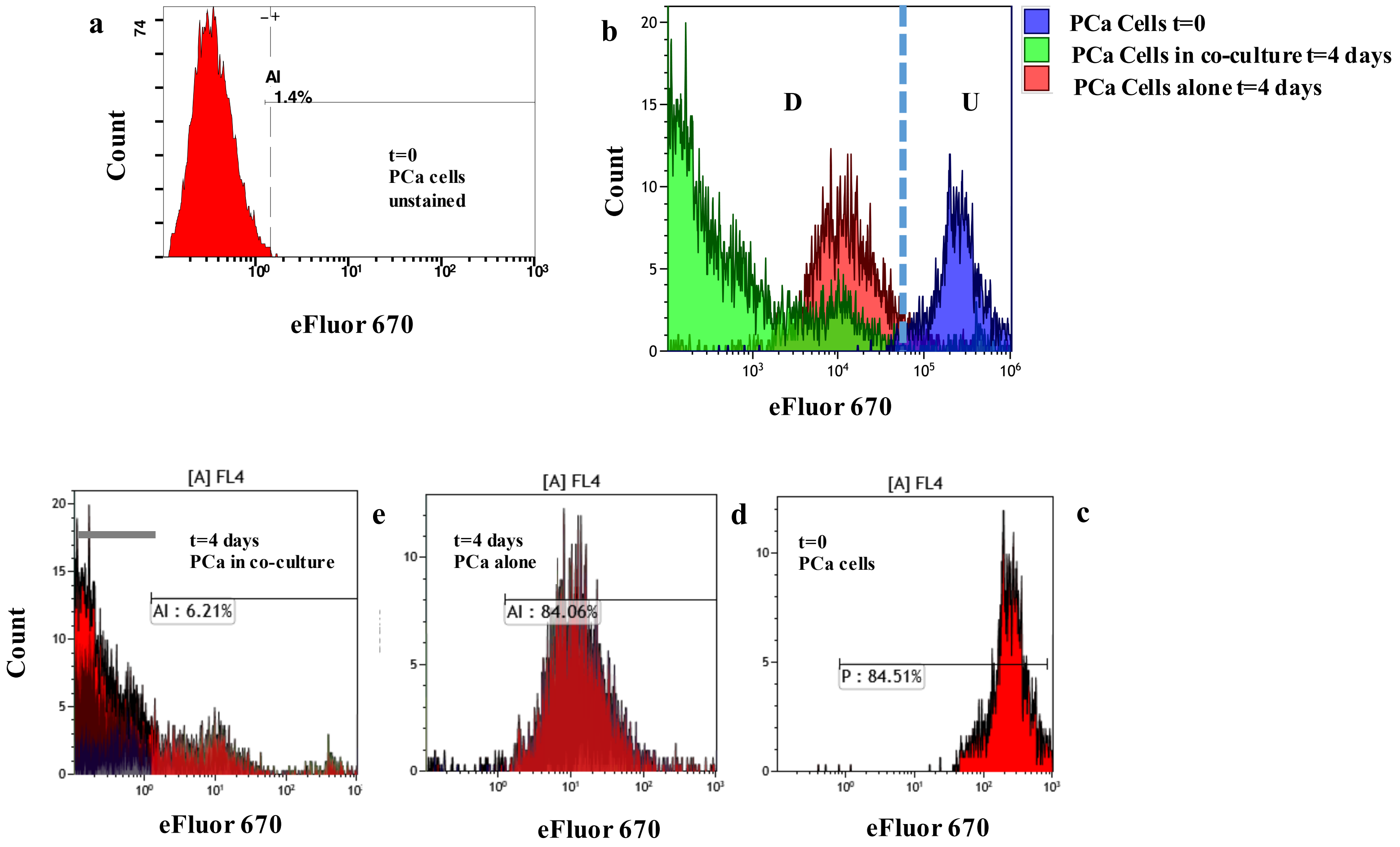

Supplementary Figure 5: eFluor 670-labeled PCa cells proliferation in co-culture with 3D bone tissues. (a) Unstained PCa cells. (b) Composite histogram plot. (c,d,e) Individual histogram plots. U=undivided (at time = 0) and (D) divided cells (4 days post-culture). Gray bar on (d) indicates non-eFluor 670+ cells (i.e., osteocytic cells).

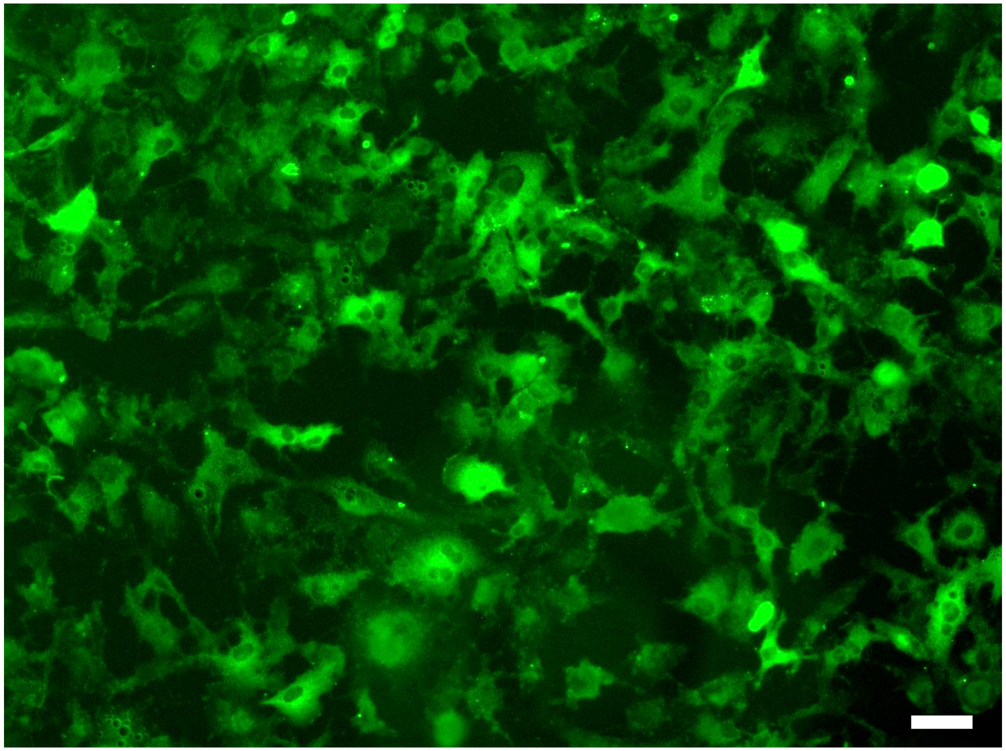

Supplementary Figure 6: Pan-cytokeratin staining of CR PCa cells. Scale bars = 50 µm.
